# Preoperative planning of calcium deposit removal in calcifying tendinitis of the rotator cuff - possible contribution of computed tomography, ultrasound and conventional X-Ray

**DOI:** 10.1186/1471-2474-15-385

**Published:** 2014-11-20

**Authors:** Kaywan Izadpanah, Martin Jaeger, Dirk Maier, Norbert P Südkamp, Peter Ogon

**Affiliations:** Department of Orthopaedic and Trauma Surgery, University Hospital Freiburg, Hugstetter 55, 79106 Freiburg im Breisgau, Germany; Center of Orthopedic SportsMedicine Freiburg, Freiburg, Germany

**Keywords:** Calcifying tendinitis, Computed tomography, Ultrasound, Consistency, Preoperative planning

## Abstract

**Background:**

The purpose of the present study was to investigate the accuracy of Ultrasound (US), conventional X-Ray (CX) and Computed Tomography (CT) to estimate the total count, localization, morphology and consistency of Calcium deposits (CDs) in the rotator cuff.

**Methods:**

US, CX and CT imaging was performed pre-operatively in 151 patients who underwent arthroscopic removal of CDs in the rotator cuff. In all procedures: (1) total CD counts were determined, (2) the CDs appearance in each image modality was correlated to the intraoperative consistency and (3) CDs were localized in their relation to the acromion using US, CX and CT.

**Results:**

Using US158 CDs, using CT 188 CDs and using CX 164 CDs were identified. Reliable localization of the CDs was possible with all used diagnostic modalities. CT revealed 49% of the CDs to be septated, out of which 85% were uni- and 15% multiseptated. CX was not suitable for prediction of CDs consistency. US reliably predicted viscous-solid CDs consistency only when presenting with full sound extinction (PPV 84.6%) . CT had high positive and negative predictive values for detection of liquid-soft (PPV 92.9%) and viscous-solid (PPV 87.8%) CDs.

**Conclusion:**

US and CX are sufficient for preoperative planning of CD removal with regards to localization and prediction of consistency if the deposits present with full sound extinction. This is the case in the majority of the patients. However, in patients with missing sound extinction CT can be recommended if CDs consistency of the deposits should be determined. Satellite deposits or septations are regularly present, which is of importance if complete CD removal is aspired.

**Electronic supplementary material:**

The online version of this article (doi:10.1186/1471-2474-15-385) contains supplementary material, which is available to authorized users.

## Background

Ultrasound (US) and Conventional X-Ray (CX) of the shoulder can be considered as standard modalities for diagnosing calcifying tendinitis of the rotator cuff. Moreover, they are used for preparation of calcium deposit (CD) removal. Preparation for any kind of invasive procedure should estimate the localization, the total number, the morphology and the consistency of the CDs. Estimating the CDs consistency seems especially relevant to choose the best modality for CD removal. Liquid-soft CDs might be removable with ultrasound guided needling [1]. Removal of viscous-solid deposits might preferably be done arthroscopically to reduce the amount of postoperative residuals [2]. Liquid-soft CDs consist of snowflake like carbonate apatite in a clear liquid, “snowstorm” pattern. Viscous-solid CDs have higher concentration of carbonate apatite and less liquids presenting like “toothpaste” after opening. Moreover, to reduce operation time and the extent of the bursectomy needed to visualize the CDs, preoperative localization is advisable [3]. In addition, the knowledge about the CDs shape might help the treating physician to dissolve the carbonatapatite away from the tendon. However, so far there exist little knowledge about the value of both US and CX to correctly estimate the above-mentioned criteria. Moreover, Farin et al. [4] reported computed tomography to add some information for CD characterization. However, it is not routinely performed in all institutions.

Aim of the present study therefore was to investigate the value of CX, US and CT for predicting localization, morphology, the total number and the consistency of CDs in the rotator for preparation of removal. We hypothesized, that CX and US might not be sufficient for thorough preoperative preparation in all cases.

## Methods

### Patient selection

A retrospective investigation was carried out on all patients that received arthroscopic calcium deposit removal between 2008 and 2012. All patients that preoperatively received the full standard investigation protocol of the senior authors affiliation, including US, CX and a CT-Scan of the shoulder before an arthroscopic CD removal were included into the present study. Patients who failed to receive one of these diagnostic procedures due to whatever reason were excluded from the present study. The present study was approved by the local ethics committee of the University Medical Center Freiburg (Nr.:329/12).

### Ultrasound

**Figure 1:**
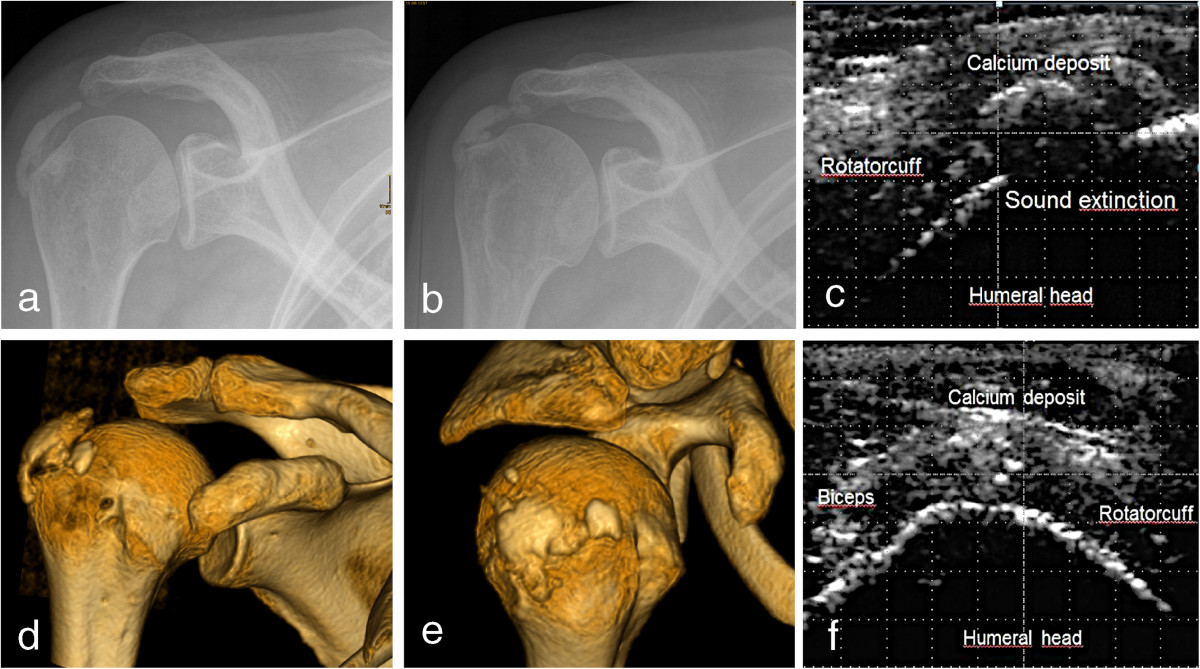
**Imaging of calcium deposit in calcifying tendinitis with conventional X-rays, ultrasound and computed tomography.** Conventional X-rays **(a+b)**, ultrasound **(c+f)** and computed tomography **(d-e)** of the same patient suffering a calcifying tendinitis of the rotator cuff. The Ultrasound image of the patient presented with full sound extinction **(c)**. Underneath a CD without sound extinction is shown **(f)**.

All patients were investigated preoperatively by a single investigator and in a standardized way (longitudinal and transverse views). The patient was sitting on a stool and the arm hanging down relaxed in a neutral position. Examination included the investigation of the rotator cuff, the localization of the long head of the biceps tendon, a localization of the calcium deposit with respect to the acromion according to the quadrant technique of Ogon and Coworkers [5] was carried out. Using this technique the Acromion was divided into 4 equal intercepts in anteroposterior direction. Moreover documentation of sound extinction (complete, incomplete or none) [6] (Figure 1) was carried out. Presence or absence of sound extinction was used for estimation of the CDs consistency. Full sound extinction was expected to correlate with a higher amount of carbonatapatite and higher density of the CD.

### Conventional X-Ray

All patients underwent a conventional radiographic examination consisting of a “true ap view” in internal and external rotation (Figure 1) and an outlet views. About 52% of the images were obtained in the seniors authors institution. Images were evaluated during the present study by the senior author and calcific deposits were classified according to the Gärtner classification scheme in order to estimate the consistency of the CDs [7]. Dense CDs were expected to have a higher amount of carbonatapatite and therefore higher density. Calcium deposits were localized according to Ogon and coworkers [8]. Sectors were defined in the outlet view. The acromion was divided in Sectors 1-3 and the region posterior to the acromion in Sector 4. Only patients imaged at our institution were included in this subgroup analyzes in order to exclude the influence of projections as images from other institutions were not taken with the same standard.

### Computed tomography

All patients included into the study received a CT scan using a dual-source-64-channel multi-detector-computed-tomography-scanner (Somatom-Defintion, Siemens, Germany) of the affected shoulder. A 3D dataset was obtained from the acromioclavicular joint to the diaphysis of the humerus (Protocol: 120 kV, 143 mA, TI 1 s, cSL 0,6mm). Image post-processing was carried out using the medical DICOM viewer (IMPAX 6, Agfa HealthCare NV, Belgium). Total count of the deposits was determined. In case of multiple deposits small ones with a distance <5 mm to a larger deposit were defined as satellite deposits. 3D reconstructions were used for determination of the morphology of the CDs looking for septations. In all cases the average Hounsfield units of the calcium deposit was determined. In case of multiple localizations the biggest deposit was chosen for investigation. Using the 3D Reconstruction also the localization of the calcium deposits was carried out. Therefore the acromion was also divided into 4 equal sectors, according to the technique from Ogon et al, used for the sonographic investigation (Figure 1).

### Surgical procedure

**Figure 2:**
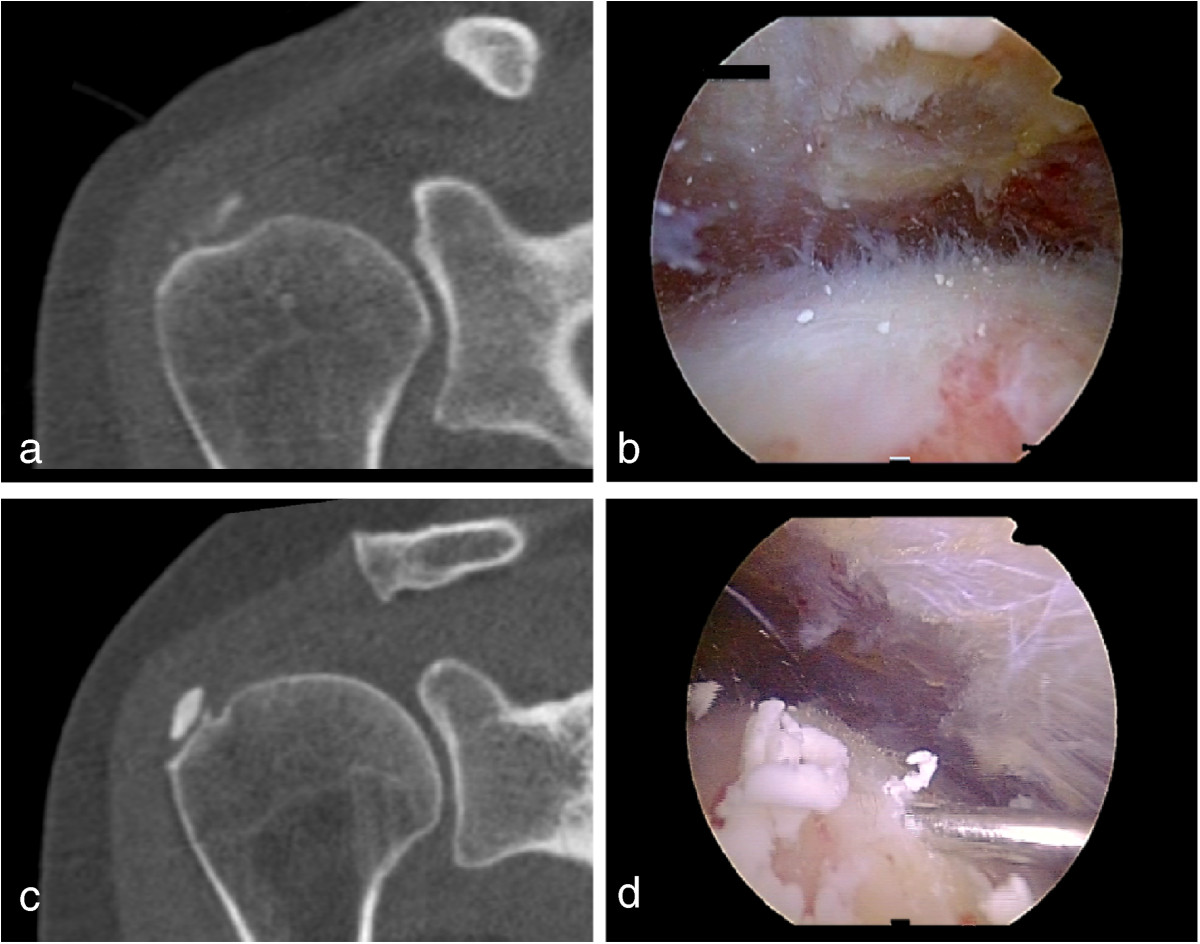
**CT scans with corresponding intraoperative findings.** In the left row CT scans of a patient with a liquid-soft deposit **(a)** and with a viscous-solid deposit **(c)** are displayed. In the right row the corresponding intraoperative findings are displayed (liquid-soft deposit **(b)**, viscous-solid deposit **(d)**.

All operative procedures were carried out by the senior author of the present manuscript in a lateral decubitus position. Careful attention was given to assure a neutral arm rotation as during CT an US examination. The bony landmarks of the shoulder girdle and the four quadrants of the acromion were drawn onto the skin. In all patients a diagnostic glenohumeral arthroscopy was performed in order to look for intraarticular pathologies through a posterior standard portal. Afterwards the scope was placed in the subacromial space and a lateral approach was created according to the preoperative estimated quadrant. A bursectomy was performed in the region underneath. Afterwards an 18 gauge spinal needle was used to penetrate the suspected region until the deposit was found (Figure 2). It was documented if the deposit could be found within the suspected region or an additional skin incision had to be performed. After Identification of the deposit a hook probe was inserted into the CD (Figure 2). In most cases a typical “toothpaste” appeared. In other cases the erupted carbonatapatite appeared more like a “snowstorm”. The hook was then used as a “stir” to remove the carbonate apatite within the deposit (Figure 2). Afterwards the superior CD membrane was elevated with the probe to enable fluid to enter the deposit. Additionally the CD was expressed by application of pressure from the bursal side (“Squeeze and stir”). In case of satellite or additional deposits they were searched and removed depending on the size either by needling or the above described method. During the procedure no imaging modalities were used.

### Statistical analyzes

Calculation of the variables were calculated using the Pearson-Test. Correlations was defined as very strong if the r value was >0.8; moderate from 0.60–0.79, fair from 0.30–0.59 and weak below 0.29. Statistical significance was assumed at p< .05. Calculation of the sensitivity, specifity and the positive and negative predictive value were calculated for US, CT and CX using a 2x2 table on the base of the intraoperative findings of the CDs texture.

## Results

In 151 patients US, CX and a CT were carried out preoperatively. Removal of the calcium deposits was possible through the lateral approach that was planned on the base of the Ogon sectors US in all patients. Intraoperative consistency of the CDs was viscous-solid in 72.8% and soft-liquid in 27.2%. The senior author performed description and graduation of the CDs texture in all cases. Two Groups were formed based on these descriptions. Group 1 (G1) included all patients with soft-liquid CDs and Group 2 (G2) included all patients with viscous-solid CDs.

**Figure 3:**
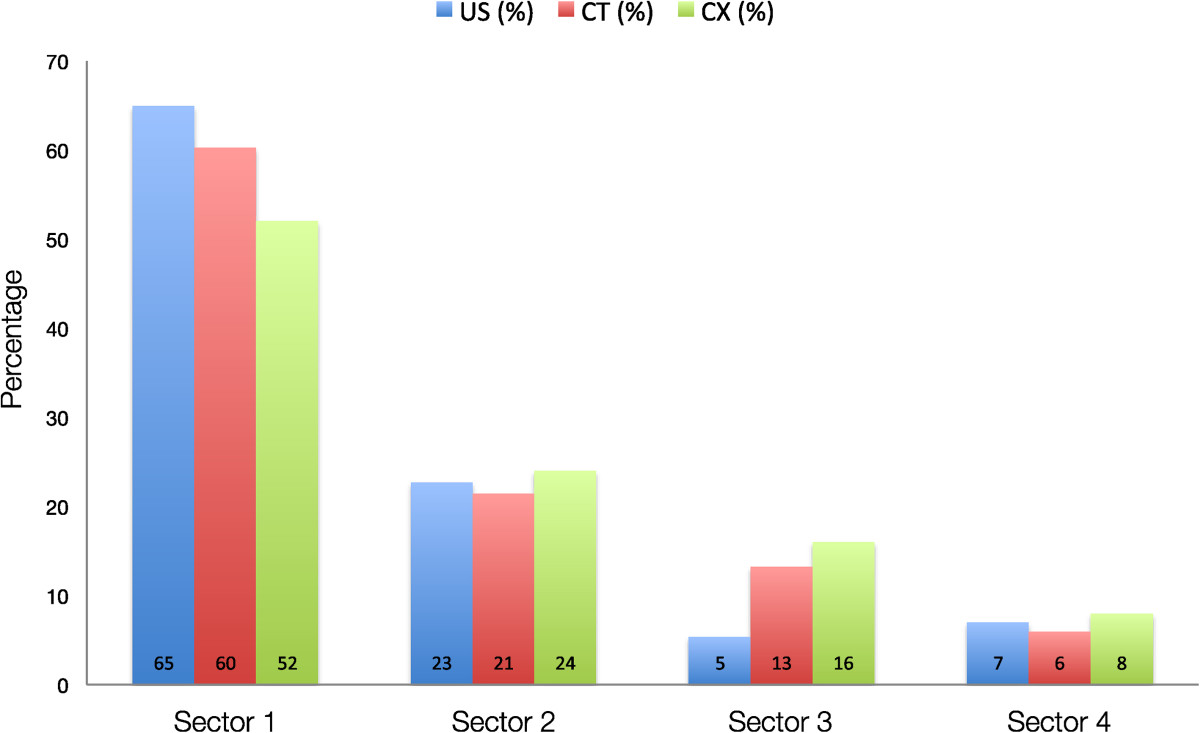
**Distribution of CDs in Sectors of the Quadrant Technique using US, CT and CX for localization.** Local distribution of calcium deposits in the rotator cuff, according to the Ogon classification. Distribution is displayed in a Barchart as a percentage of the total amount, found by each investigated imaging technique (blue-US, red- CT, green –CX).

Differences were found determining the total number of calcium deposits. Using Ultrasound 158 CDs, using Computed Tomography 188 CDs and using Conventional X-rays 164 CDs were identified. With US in 95.4% of the patients one deposit was documented and in 4.6% 2 deposits. With CT in 78.8% of the patients one deposit was documented, in 18.5% 2 deposits, in 2% 3 deposits and in 0.7% 4 deposits. With CX In 94% of the patients one deposit was documented, in 4% 2 deposits, in 1.3% 3 deposits and in 0.7% 4 deposits.The Distribution of CDs to the sectors was mainly coherent over the image modalities (Figure 3). The majority of deposits were found within the Sector 1 and 2. However, it has to be pointed out that only 52% of all patients had a standardized outlet view at the senior authors institution with standardized positioning. Only these patients were included in this subgroup analyzes in order to exclude the influence of projections for the investigation from other institutions. In 17 patients (11%) an additional deposit was identified using CT and in 6 of these (35%) during operation an additional deposit in another quadrant was removed, requiring extra bursectomy.

A difference in the ability to predict the consistency of the CDs was found. With ultrasound a fair correlation of sound extinction with the intraoperative texture was found (k= 0.589, p<0.001 Pearson). The average CT density of the CDs (Hounsfield units) moderately correlated with the intraoperative texture of the CDs (k= 0.603. p<0.001 Pearson). The Gärtner classification showed only poor correlation with the intraoperative consistency (k= -0.359, p<0.001 Pearson). The positive and negative predictive value (PPV, NPV) of US, CT and CX to correctly estimate the CDs texture were calculated. Moreover, the sensitivity and specifity to detect liquid-soft and viscous-solid CDs were calculated. The NPV and PPV values were moderate (Table 1). CT showed very high sensitivity for detection of viscous-solid CDs with moderate specifity. Moreover high PPV and NPV were found for liquid-soft and vicious-solid deposits (Table 2). Using x-Ray sensitivity and specifity to detect both, solid and liquid CDs was low. PPV and NPV to predict a solid or a vicious CD were only moderate to poor (Table 3).

**Table 1:** **Total counts of patients grouped according to the intraoperative CD consistency and CT density**

CT		Soft/Liquid (n)	Solid(n)	n	
TP	HU <300 (n)	26	2	28	PPV _Soft/Liquid_/NPV_Solid_ 92.9
TN	HU >300 (n)	15	108	123	PPV _Solid_/NPV_Soft/Liquid_ 87.8
		41	110	151	
	Specifity	98.2	63.4		
	Sensitivity	63.4	98.2		

Specifity and sensitivity to diagnose a soft or solid deposit and positive (PPV and negative (NPV) predictive value of the CTs have been calculated.

**Table 2:** **Total counts of patients grouped according to the intraoperative CD consistency and the according sound extinction**

US		Soft/Liquid (n)	Solid (n)		
TP	No Sound Extinction (n)	22	4	26	PPV _Soft/Liquid_/NPV_Solid_ 84.6
TN	Sound Extinction (n)	19	106	125	PPV _Solid_/NPV_Soft/Liquid_ 84.8
		41	110	151	
	Specifity	96.4	53.7		
	Sensitivity	53.7	96.4		

Specifity and sensitivity to diagnose a soft or solid deposit and positive (PPV and negative (NPV) predictive value of the USs have been calculated.

**Table 3:** **Total counts of patients grouped according to the intraoperative CD consistency and the according Gärtner classification**

CX		Soft/Liquid	Solid		
TP	Gärtner 3	16	10	26	PPV _Solid_/NPV_Soft/Liquid_ 61.5
TN	Gärtner 1+2	25	100	125	PPV _Solid_/NPV_Soft/Liquid_ 80.0
		41	110	151	
	Specifity	90.9	39.0		
	Sensitivity	39.0	90.9		

Specifity and sensitivity to diagnose a soft or solid deposit and positive (PPV) and negative (NPV) predictive value of the CXs have been calculated.

Investigating the morphology of the CDs CT revealed that 49% of all deposits had septations. From these 85% were uni- and 15% multiseptated. Septations could not be detected with US. Using CX Septations were seen suspected in 26,5% of all patients. Additional with CT a cystic lesion of the humeral head with connection to a calcium deposit was present in 21%.

## Discussion

Calcifying tendinitis of the rotator cuff is a frequent disease of the shoulder with an estimated prevalence of 3% [9]. About 30% of these individuals turn symptomatic once in their lifetime [9]. The majority of them can be treated conservatively. However, about 15% require invasive treatment [10]. Different modalities have been developed over time to remove CDs from the rotator cuff, i.e. Needling [3], Barbotage [1] or Arthroscopic CD [2, 11, 12] removal. These procedures are reported to have heterogeneous clinical outcomes (Constant-Score ranging from 74-100 points). Complete CD removal rates after Follow up are reported to be higher after arthroscopic removal than after needling (92% [11–14] vs. 47% [6, 7, 15, 16]). Possible explanations might be that solid CDs are less responsive to needling than to arthroscopic removal. Moreover, CDs medial to the acromion cannot be displayed easily with ultrasound. Therefore prediction of the CDs consistency and preoperative localization seems important in order to choose the best treatment modality.

**Figure 4:**
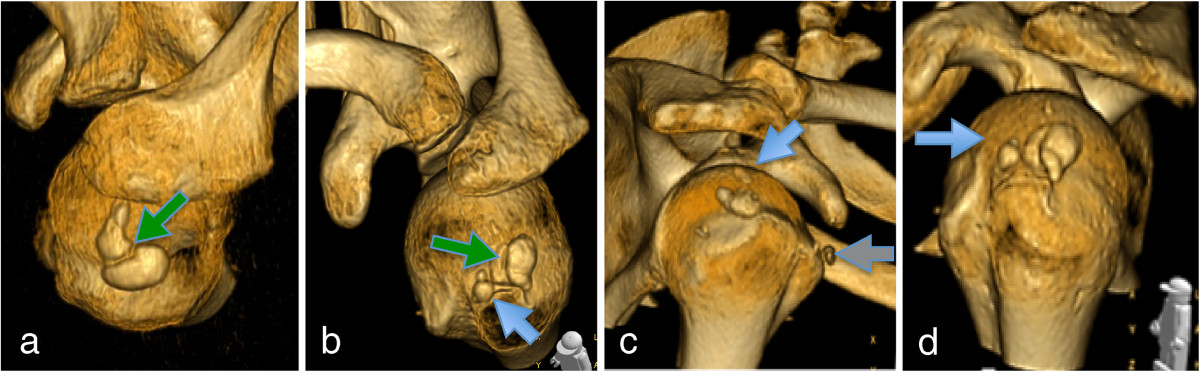
**3D Reconstruction of CT-Scans from patients suffering from a calcifying tendinitis of the rotator cuff.** Green arrows indicate septations of the deposit **(a+b)**. Blue arrows display satellite deposits that are associated to a larger deposit but have no more distance than 5mm **(b, c+d)**. The grey arrow indicates an additional deposit in an alternative quadrant **(c)**.

**Figure 5:**
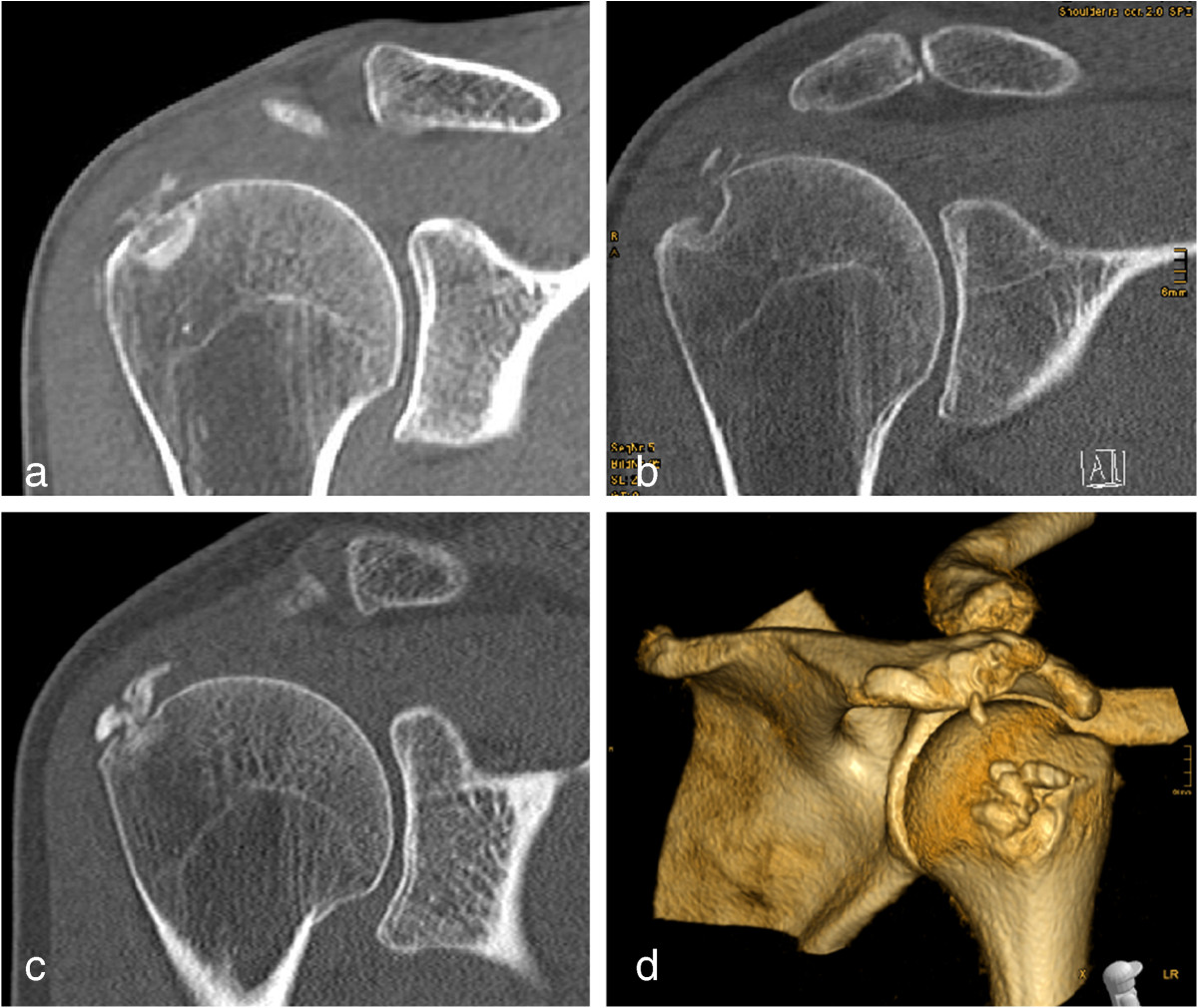
**CT scans (a-c) and a 3 D reconstruction (d) showing humeral cysts developing from CD.**

CX and US are well accepted as modalities for diagnosing calcifying tendinitis of the rotator cuff. However, there is little knowledge about their true value to characterize CDs for preparation of invasive deposit removal. Farin et al. [4] reported one of the view studies that compared US, CX and CT with findings during rotator cuff needling for calcium deposit removal. In total they investigated 58 patients and 61 cases out of which 20 cases received a CT scan in addition to US and CX of the shoulder. They found only a moderate correlation between sound extinction at US and densities at CT (Hounsfield units) with the CDs consistency. In this study plain radiographs seemed not suitable for the prediction of the CDs consistency. They concluded, that US is a suitable method for the selection of patients for needle treatment. Despite the reliable scientific setup of the study just a rather small population of 20 patients were investigated with all three imaging modalities and a selection bias has to be stated, as all patients were found suitable for needling as a treatment of choice before recruiting them for the study. Moreover, morphologic findings of the CDs during preoperative evaluation were not described in detail. In the present study, a larger cohort of 151 patients was investigated. Arthroscopic deposit removal was performed in all investigated patients and enabled direct visualization of the CDs for evaluation of their true consistency, count and localization. These findings were compared with the data from the preoperative CX, US and CT. Localization of calcium deposits during operative treatment can be demanding and several techniques have been proposed to facilitate this action [17, 18]. In the present study, prediction of the CD localization with local relation to the acromion was possible in all cases using US with the technique described by Ogon et al. [5]. The transformation of this technique to CT and CX showed a quasi-identical distribution of the CDs except a slight higher number of CDs in the third Sector. This might be explained by the additional CDs detected with CX and even more with CT. All methods therefore seem suitable for localization of the CDs. Moreover CT identified septations (Figure 4) in nearly half of all cases and a humeral cyst was found in 21 % (Figure 5). The septations were not reliably detectable with either CX or US, however they are more likely to be present in cases of an irregular shape of the CD in CX or US. Looking into the recent literature such septations or satellite deposits have not been discussed adequately. Therefore, the true incidence of these phenomena (including patients successfully treated conservatively) cannot be estimated. However, there exists a relevant amount of these cases in patients from the present study and the treating surgeon should be aware of this as they influence the treatment needed. Especially in those techniques taking an effort for rotator cuff preservation like needling, barbotage or the one performed in the present study, “Squeeze-and-Stir” it is important to open all portions of the CD. Moreover if a satellite lesion can be found it has to be addressed separately. As a conclusion, if a CT was not carried before arthroscopic procedure the authors have started to perform a circumferential needling around identified CDs under vision to perforate possible septations or needle small satellite deposits. It has to be pointed out that the need of complete intraoperative removal of the CDs might not be necessary as several studies show that either secondary resorption of the deposits develop or there exists no direct correlation between removal and clinical outcome [15, 19–22]. However, the authors believe penetration of the CDs might propagate postoperative resorption of deposits. Apart from these morphologic parameters, with the present study, US were found suitable for the estimation of the CDs consistency in deposits with a full sound extinction. However, US is not useful to predict the deposits consistency in case of missing sound extinction. CT was found to have high positive and negative predictive values for estimation of both soft-liquid and viscous-solid CDs. Prediction of the Consistency with conventional CX according to the Gärtner classification was not reliably possible. CT is therefore superior to the other methods estimating the consistency of the CDs. However, in the hands of an experienced physician CT is just needed besides US in the case of absent sound extinction. Overall CT offers the greatest abilities to predict all the investigated parameters. Greater financial resources needed and the higher exposure to radiation for patients are reasons to not recommend CT in all cases. However, advances of the latest CT-Scanners and post processing techniques enable drastic reduction of the radiation exposure [23]. This might lead to a broader use of CT Scans in the future.

The present study suffers some limitations. At first, all patients have been treated in a single hospital and therefore a selection bias cannot be excluded. At second CX views were not all performed in the same hospital and views therefore were not standardized in all cases, which led to the Exclusion of some patients.

## Conclusions

US and CX are sufficient for preoperative planning of CD removal with regards to localization and prediction of consistency if the deposits present with full sound extinction. This is the case in the majority of the patients. However, in patients with missing sound extinction CT can be recommended if CDs consistency of the deposits should be determined. Satellite deposits or septations are regularly present, which is of importance if complete CD removal is aspired.

## Authors’ original submitted files for images

Below are the links to the authors’ original submitted files for images. Authors’ original file for figure 1 Authors’ original file for figure 2 Authors’ original file for figure 3 Authors’ original file for figure 4 Authors’ original file for figure 5
