# Supplementary figures and images for: Preoperative planning of calcium deposit removal in calcifying tendinitis of the rotator cuff - possible contribution of computed tomography, ultrasound and conventional X-Ray
Source: BMC Musculoskelet Disord. 2014 Nov 20;15:385. doi: 10.1186/1471-2474-15-385 (PMC4246434; doi:10.1186/1471-2474-15-385)

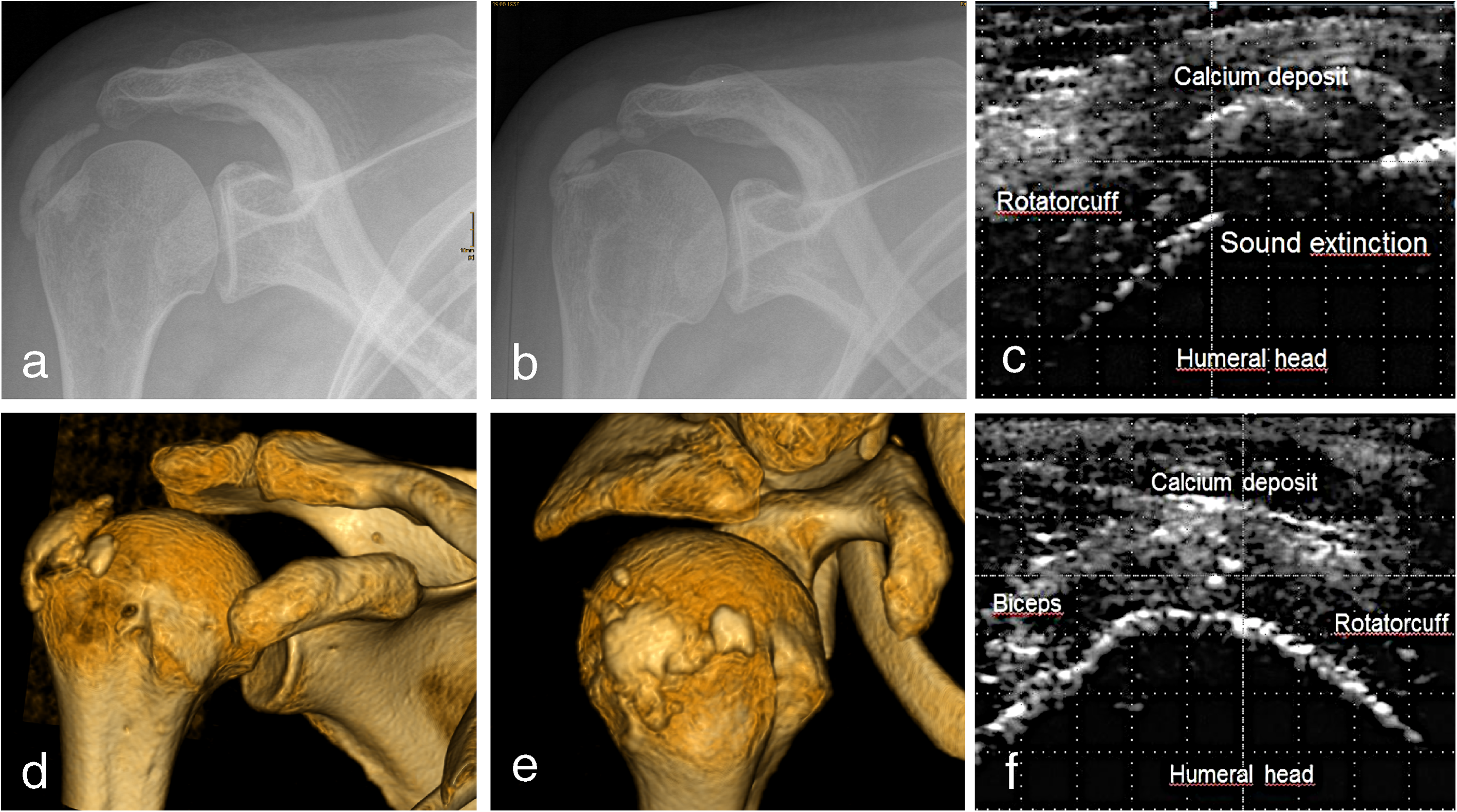

Supplement: Supplementary file 1 — Authors’ original file for figure 1 [file 12891_2014_2310_MOESM1_ESM.tif]

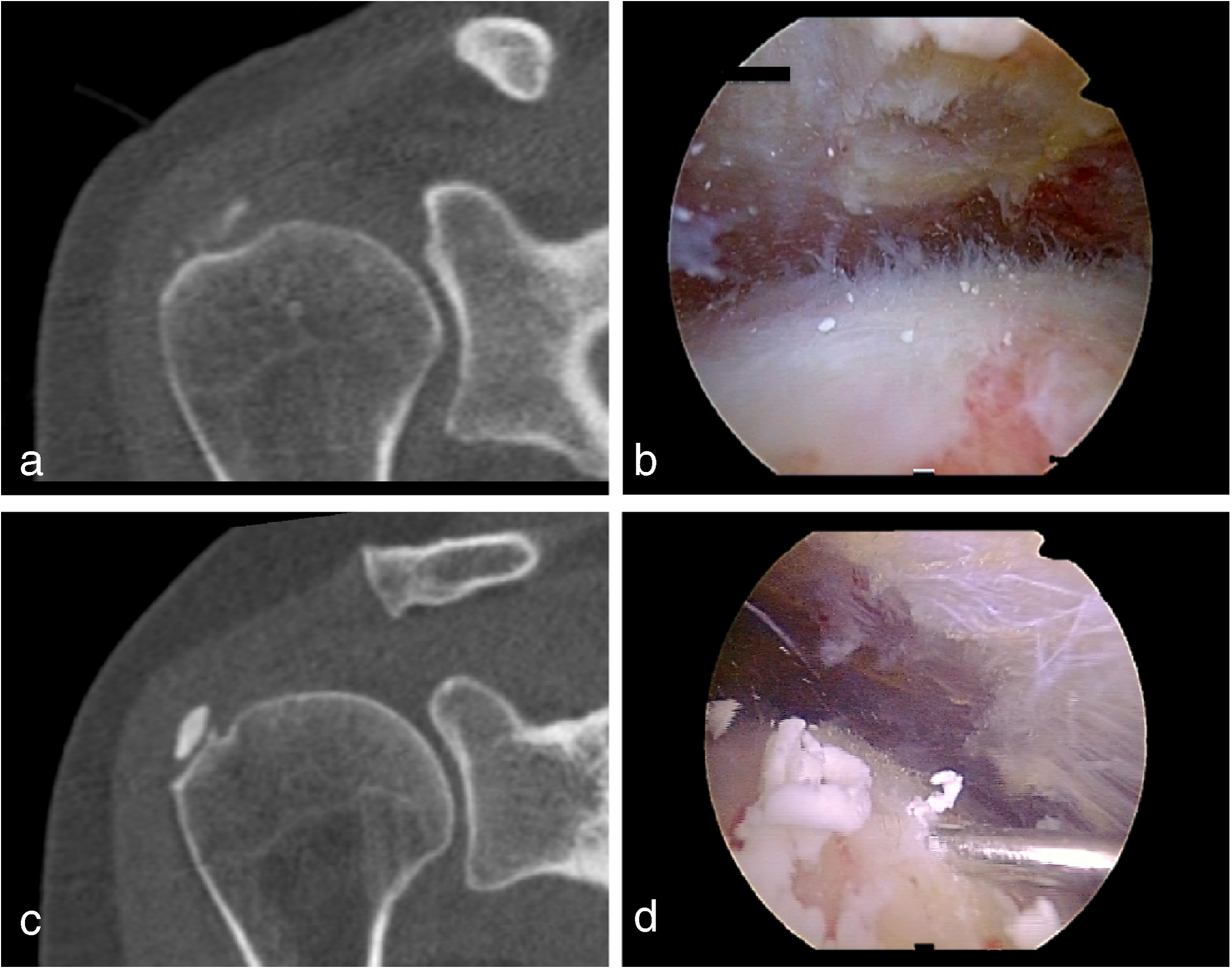

Supplement: Supplementary file 2 — Authors’ original file for figure 2 [file 12891_2014_2310_MOESM2_ESM.tif]

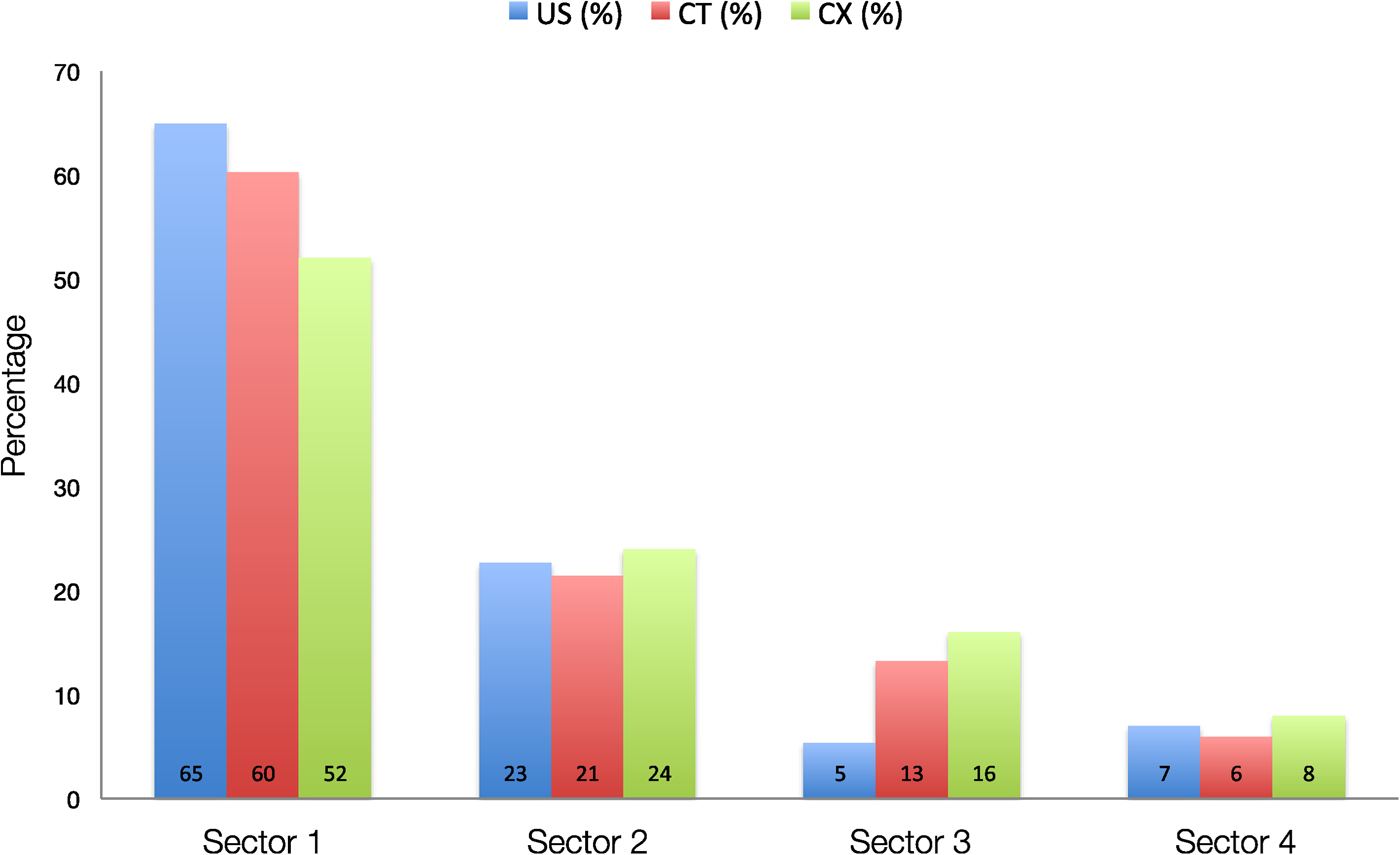

Supplement: Supplementary file 3 — Authors’ original file for figure 3 [file 12891_2014_2310_MOESM3_ESM.tif]

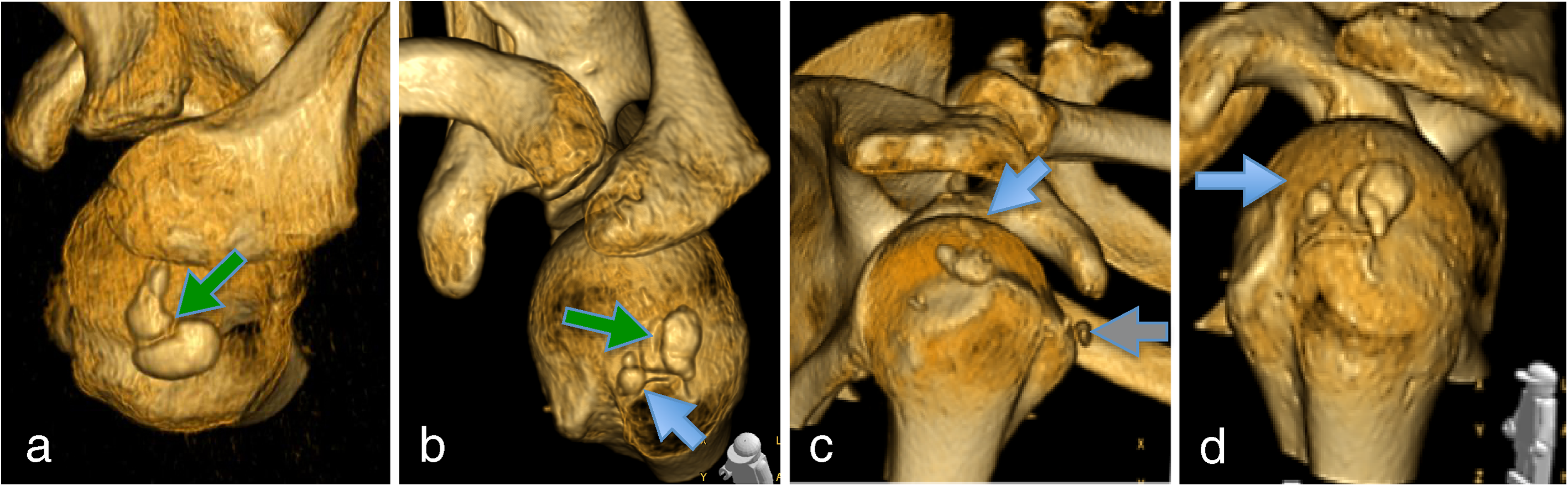

Supplement: Supplementary file 4 — Authors’ original file for figure 4 [file 12891_2014_2310_MOESM4_ESM.tif]

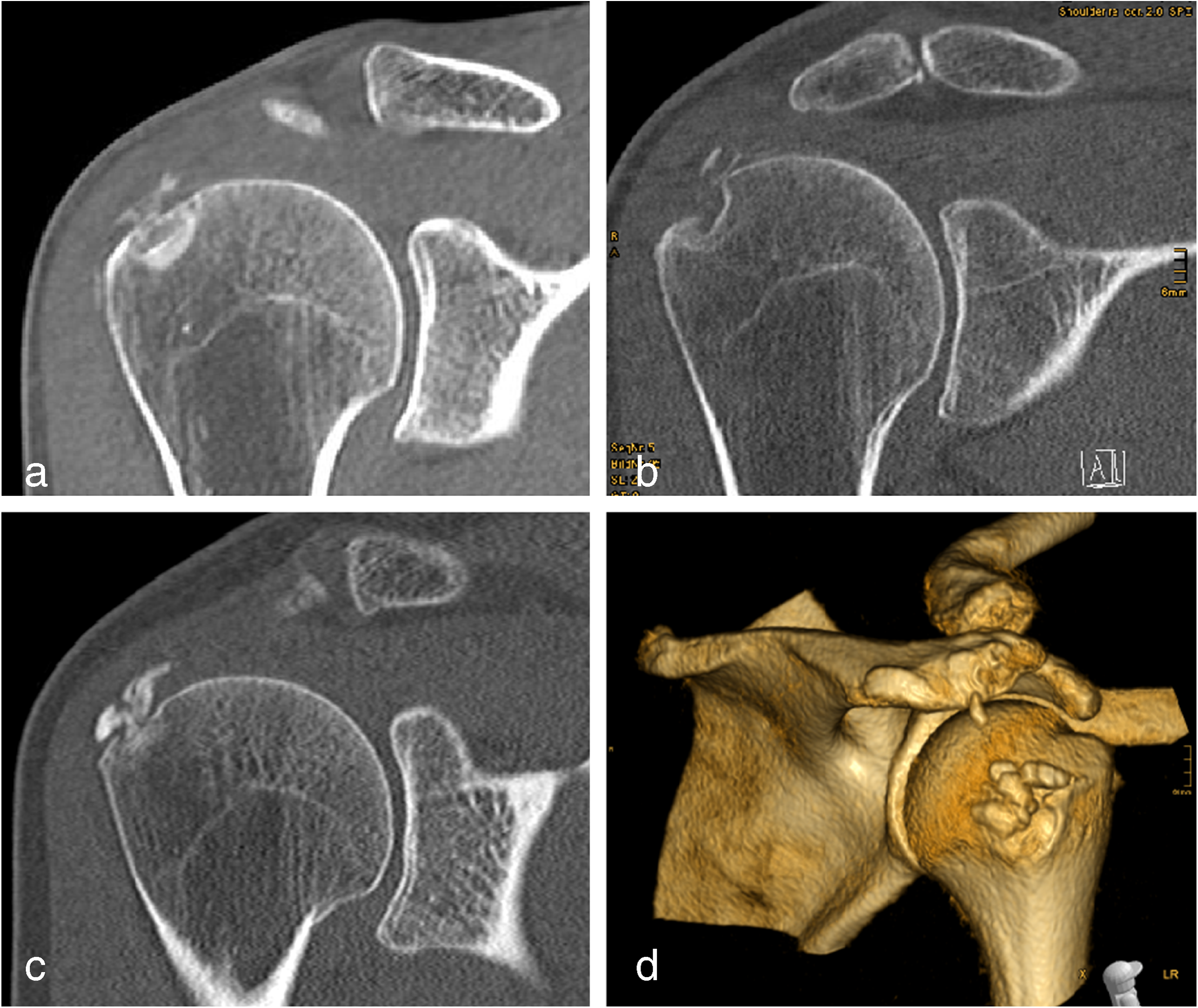

Supplement: Supplementary file 5 — Authors’ original file for figure 5 [file 12891_2014_2310_MOESM5_ESM.tif]
